# In Silico and Molecular Docking Analysis of Benzyl Isothiocyanate from *Salvadora persica* as a Predicted Multi-Target Candidate for COVID-19 Host Responses

**DOI:** 10.3390/cimb48080849

**Published:** 2026-08-21

**Authors:** Terrence Suministrado Sumague, Ibrahim M. Aziz, Reem M. Aljowaie, Asma N. Alsaleh, Noorah A. Alkubaisi, Fahad N. Almajhdi

**Affiliations:** 1 Molecular and Cell Biology Laboratory, Prince Naif bin AbdulAziz Health Research Center, College of Dentistry, King Saud University, P.O. Box 60169, Riyadh 11545, Saudi Arabia; tsumague@ksu.edu.sa; 2 Department of Botany and Microbiology, College of Science, King Saud University, P.O. Box 2455, Riyadh 11451, Saudi Arabia; iaziz@ksu.edu.sa (I.M.A.); raljowaie@ksu.edu.sa (R.M.A.); asmalsaleh@ksu.edu.sa (A.N.A.); nalkubaisi@ksu.edu.sa (N.A.A.)

**Keywords:** COVID-19, *Salvadora persica*, miswak, benzyl isothiocyanate, bioinformatics, network pharmacology, molecular docking

## Abstract

COVID-19 remains a relevant area of biomedical investigation because its pathogenesis involves complex virus–host interactions. This study aimed to explore, through purely in silico and hypothesis-generating insights, the predicted molecular associations between benzyl isothiocyanate (BITC) from *Salvadora persica* and COVID-19-associated host-response pathways. BITC-associated targets were collected from compound-target databases, while COVID-19-associated targets were obtained from disease-gene databases and transcriptomic datasets. Overlapping targets were analyzed using protein–protein interaction network construction, hub-gene prioritization, Gene Ontology and KEGG enrichment analyses, and molecular docking. A total of 271 unique BITC-associated targets and 1890 COVID-19-associated targets were identified, with 36 candidate targets overlapping. PPI analysis generated a connected network of 24 nodes and 39 edges. Hub-gene analysis prioritized ACE, JUN, MAOA, CDK1, MAOB, HCK, CCNA2, ACHE, GADD45A, and ADRA2A. Enrichment analysis indicated associations with inflammatory response, vascular regulation, calcium homeostasis, monoamine oxidase activity, NF-κB signaling, serotonergic synapse, and tryptophan metabolism. Validated active-site docking of six targets yielded comparative Vina scores ranging from −5.380 to −6.437 kcal/mol. These preliminary findings provide theoretical target–pathway associations supporting further investigation of BITC as a potential immunomodulatory candidate within COVID-19-related host-response pathways.

## 1. Introduction

Coronavirus disease 2019 (COVID-19), caused by severe acute respiratory syndrome coronavirus 2 (SARS-CoV-2), remains a relevant area of biomedical investigation because its pathogenesis involves complex virus–host interactions, variable clinical outcomes, and post-acute sequelae [1,2,3]. The World Health Organization (WHO) continuously monitors COVID-19 cases and has reported approximately 779 million cumulative cases worldwide, although current epidemiological estimates are influenced by changes in testing practices and reporting variability [4]. Despite advances in vaccination that have reduced severe disease outcomes, the continued evolution of SARS-CoV-2 and post-COVID-19 syndromes support further investigation of COVID-19-associated molecular mechanisms [3,5,6]. COVID-19 pathogenesis reflects interactions between viral processes and host responses, including inflammatory signaling and immune dysregulation, particularly in immunocompromised patients or those with severe disease [7,8,9]. This biological complexity provides a rationale for exploratory studies that assess virus-associated and host-response-related molecular targets without assuming therapeutic efficacy or biological activity [10].

Natural products offer structurally diverse small molecules that support preliminary compound prioritization and predictive mechanistic hypothesis generation [11]. *Salvadora persica* L., also known as *miswak*, has been widely studied for its traditional oral-hygiene use and phytochemical composition [12,13]. Benzyl isothiocyanate (BITC) is a well-documented chemical constituent of *S. persica* and has been identified and quantified in extracts and related formulations in several analytical studies [14,15,16]. Recent studies have shown the antimicrobial, antibiofilm, anti-inflammatory, and cancer-related bioactivities of BITC and isothiocyanate-containing systems in non-COVID-19 experimental contexts [14,17,18,19,20]. These findings provide a limited background rationale for exploring BITC as a potential immunomodulatory agent in relation to COVID-19-associated host-response pathways, with all inferred associations interpreted as hypothesis-generating.

**Figure 1:**
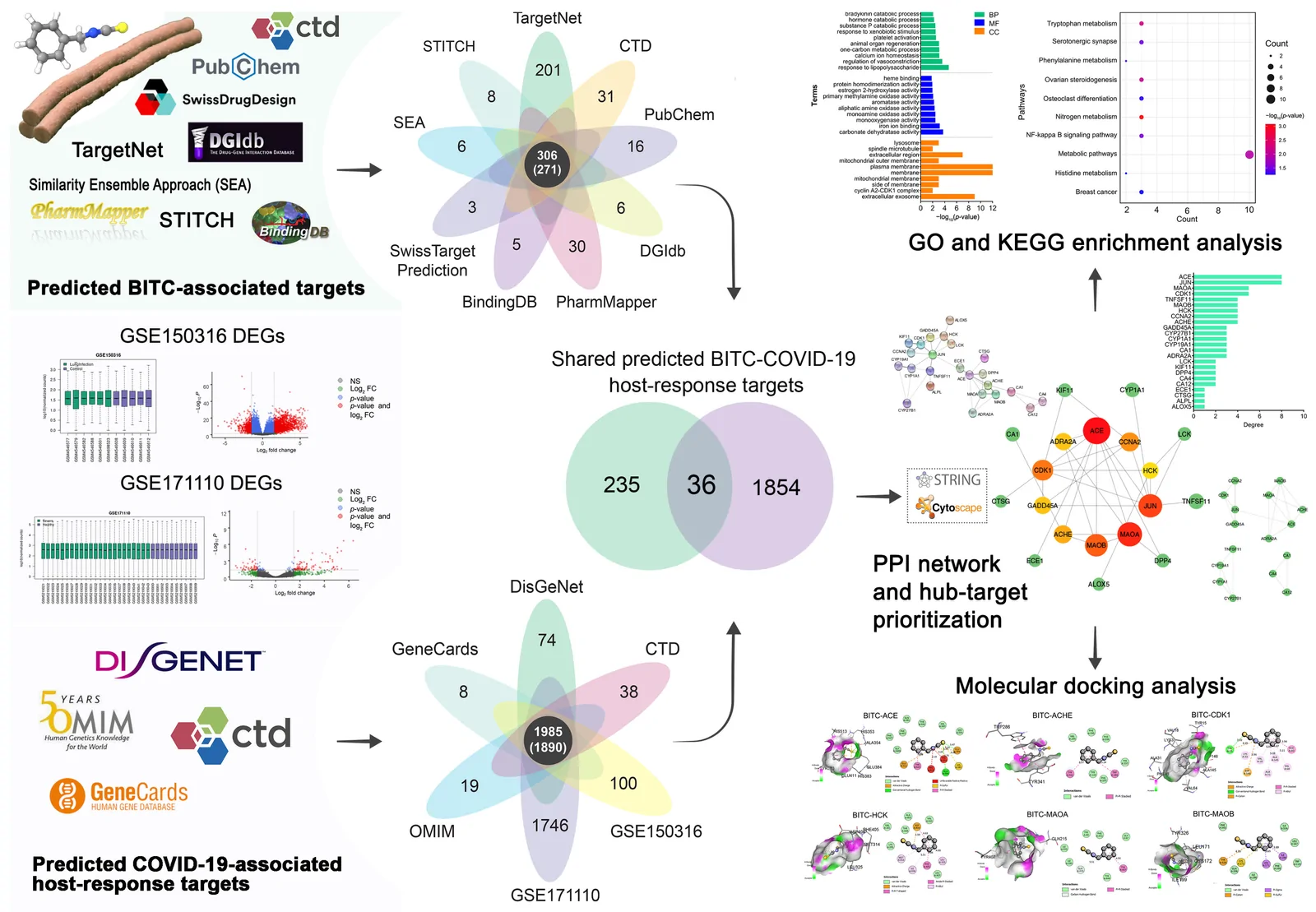
Workflow of the in silico analysis of BITC-associated targets from *S. persica* and COVID-19-related host-response pathways. Predicted BITC-associated targets were collected from compound-target databases, and COVID-19-related host-response targets were obtained from disease-gene databases and publicly available datasets. Overlapping targets were identified and subjected to protein–protein interaction network construction, hub-target prioritization, GO and KEGG enrichment analysis, molecular docking, and ADMET analysis.

Conventional compound development workflows are time- and resource-intensive, and bioinformatics-based approaches are increasingly used to support early-stage target and pathway prioritization [21]. Network pharmacology provides a system-level framework for integrating predicted compound-associated targets, disease-related genes, protein–protein interaction networks, and pathway annotations to identify potential molecular intersections for hypothesis generation [22]. Functional enrichment analysis using Gene Ontology (GO) and the Kyoto Encyclopedia of Genes and Genomes (KEGG) further supports biological interpretation by identifying over-represented gene functions and pathway categories among the selected targets [23,24]. Molecular docking is one of the major in silico tools that provides preliminary insights into candidate compounds and targets molecular structures to estimate the binding poses and affinity scores of the compounds on the target molecule [25]. This study aimed to explore the predicted molecular associations between BITC-related targets and COVID-19-associated host-response pathways using network pharmacology and bioinformatics-based analyses, as summarized in Figure 1.

## 2. Materials and Methods

### 2.1. Target Identification and Dataset Construction

#### 2.1.1. Selection and Structural Retrieval of Benzyl Isothiocyanate

Benzyl isothiocyanate (BITC) was selected as the compound of interest based on previous chemical characterization and analytical studies reporting its occurrence in *Salvadora persica* roots, extracts, and related formulations [13,14,15]. The chemical structure and canonical SMILES of BITC were retrieved from PubChem (PubChem CID: 2346; https://pubchem.ncbi.nlm.nih.gov/compound/2346) (accessed on 14 August 2025) for subsequent in silico analyses [26].

#### 2.1.2. Prediction of BITC-Associated Target

The potential BITC target genes were retrieved from multiple chemical, drug–gene interactions, and target prediction databases, including BindingDB (https://bindingdb.org/) (accessed on 14 August 2025) [27], Comparative Toxicogenomics Database (CTD; http://ctdbase.org/) (accessed on 14 August 2025) [28], Drug Gene Interaction Database (DGIdb; https://www.dgidb.org/) (accessed on 14 August 2025) [29], PharmMapper (https://www.lilab-ecust.cn/pharmmapper/) (accessed on 14 August 2025) [30], PubChem (https://pubchem.ncbi.nlm.nih.gov/) (accessed on 14 August 2025) [26], Similarity Ensemble Approach (SEA; https://sea.bkslab.org/) (accessed on 14 August 2025) [31], Search Tool for Interacting Chemicals (STITCH; http://stitch.embl.de/) (accessed on 14 August 2025) [32], SwissTargetPrediction (STP; http://www.swisstargetprediction.ch/) (accessed on 14 August 2025) [33], and TargetNet (TN; http://targetnet.scbdd.com/) (accessed on 14 August 2025) [34]. BITC-associated targets were filtered using database-specific criteria: SwissTargetPrediction probability > 0; TargetNet models with AUC ≥ 0.75, with non-zero-probability predictions; SEA *p*-value ≤ 0.0001; STITCH score ≥ 0.40; and PharmMapper fit score ≥ 0.20. Targets obtained from each database were mapped to the UniProt database with “Homo sapiens” to standardize gene symbols [35]. All BITC-associated targets were subsequently pooled, duplicates were removed, and values were integrated into the BITC-related target set.

#### 2.1.3. Identification of COVID-19-Related Targets

COVID-19-related transcriptomic targets were identified from two publicly available GEO datasets: GSE150316 [36] and GSE171110 [37] were selected to identify COVID-19-related targets from Gene Expression Omnibus (GEO) (National Center for Biotechnology Information, Bethesda, MD, USA; https://www.ncbi.nlm.nih.gov/geo/) (accessed on 21 August 2025) [38]. GSE150316 and GSE171110 were selected because they provide human bulk RNA-sequencing data from severe COVID-19 cases and non-COVID-19 controls, with clearly annotated groups and processed expression data suitable for GEO2R analysis. The datasets represent lung tissue and whole blood, respectively, and were analyzed independently. Datasets lacking suitable controls, clear sample annotations, or GEO2R-compatible expression data were excluded. For GSE150316, the selected control samples included GSM4546608, GSM4546609, GSM4546610, GSM4546611, and GSM4546612; infected lung tissue samples included GSM4546577, GSM4546579, GSM4546582, GSM4546588, and GSM4546601. For GSE171110, the healthy group included GSM5218990 to GSM5218999, whereas the severe COVID-19 group included GSM5219021 to GSM5219043. Each dataset was analyzed independently using the GEO2R RNA-sequencing workflow with count data available through GEO. Differential expression was assessed using DESeq2 with its default normalization, dispersion estimation, and statistical testing procedures [38]. Sample groups were assigned according to the metadata deposited in GEO. Because GSE150316 and GSE171110 represented lung tissue and whole blood, respectively, the datasets were analyzed separately, and their expression data were not combined. The differentially expressed genes (DEGs) between groups were analyzed, and the DEGs were selected if log2-fold change ≥ ±1.5 and adjusted *p*-value (FDR) < 0.05 after Benjamini–Hochberg correction [39]. The DEGs obtained from each dataset were visualized using RStudio (Windows version 2024.04.2+764; Posit Software, Boston, MA, USA). In addition to the GEO-derived DEGs, COVID-19-associated genes were retrieved from GeneCards [40], the Comparative Toxicogenomics Database (CTD) [28], DisGeNET [41], and Online Mendelian Inheritance in Man (OMIM) [42]. Duplicate entries were removed, and gene symbols were standardized. Subsequently, the COVID-19-related target set was intersected with the BITC-associated target set to identify candidate BITC and COVID-19-related targets for the protein–protein interaction network analysis, functional enrichment, and molecular docking.

### 2.2. PPI Network Construction of Candidate Network Hub Genes

The BITC and COVID-19 target gene lists were input into InteractiVenn (https://www.interactivenn.net/) (accessed on 28 August 2025) to identify overlapping candidate targets. The intersecting targets were then input into the Search Tool for the Retrieval of Interacting Genes database (STRING version 12.0; https://string-db.org/) (accessed on 28 August 2025) using “Homo sapiens”. The default medium-confidence interaction threshold was set to 0.4, and disconnected nodes were removed [43,44]. The resulting protein–protein interaction (PPI) network was further analyzed using through Cytoscape software (Windows version 3.10.3) [45] and the NetworkAnalyzer (version 4.5.0) to calculate the network topology; cytoHubba (version 0.1) using maximal clique centrality (MCC) algorithm to determine the top 10 ranked nodes; and Molecular Complex Detection (MCODE; version 2.0.3) to identify densely connected modules within the PPI network. The final top 10 hub genes were selected and styled according to the MCC ranking; degree centrality was used as supporting network topology information.

### 2.3. GO and KEGG Functional Enrichment Analysis

Candidate BITC-associated COVID-19 target hub genes were subjected to functional enrichment analysis using the Database for Annotation, Visualization and Integrated Discovery (DAVID) (https://david.ncifcrf.gov/) (accessed on 28 August 2025) [46]. Gene Ontology (GO) enrichment analysis was performed for biological process (BP), molecular function (MF), and cellular component (CC) categories, while Kyoto Encyclopedia of Genes and Genomes (KEGG) pathway analysis was used to identify significantly enriched pathways. The DAVID default Homo sapiens background was applied. Multiple-testing correction was assessed using the false discovery rate (FDR), with FDR < 0.05 considered statistically significant. Terms with nominal *p* < 0.05 but FDR ≥ 0.05 were retained for exploratory interpretation. The top GO terms were visualized as −log_10_(*p*-value) bar plots, and the top KEGG pathways as bubble plots showing gene count and −log_10_(*p*-value). Gene count, gene ratio, raw *p*-value, FDR, enrichment score, associated genes, and background gene universe are reported in the Supplementary Tables. Figures were generated using RStudio version 2024.04.2.

### 2.4. Molecular Docking Analysis

The three-dimensional structure of BITC was obtained from PubChem (PubChem CID: 2346) and optimized using Avogadro software (Windows version 1.2.0; https://avogadro.cc) and output to mol2 format. The molecular docking study was performed for the MCC-prioritized hub targets. The crystal structure of angiotensin-converting enzyme (ACE; PDB ID: 1O86), amine oxidase flavin-containing A (MAOA; PDB ID: 2Z5X), cyclin-dependent kinase 1 (CDK1; PDB ID: 4Y72), amine oxidase flavin-containing B (MAOB; PDB ID: 1OJ9), tyrosine-protein kinase HCK (HCK; PDB ID: 1QCF), cyclin-A2 (CCNA2; PDB ID: 7ACK), and acetylcholinesterase (ACHE; PDB ID: 8DT7) were retrieved from RCSB Protein Data Bank (RCSB PDB; https://www.rcsb.org/) (accessed on 1 September 2025) [47]. The amino acid sequence of the alpha-2A adrenergic receptor (ADRA2A; UniProt ID: P08913), DNA damage-inducible protein GADD45 alpha (GADD45A; UniProt ID: P24522), and transcription factor Jun (JUN; UniProt ID: P05412) were retrieved from UniProt database and the 3D structure was predicted using Iterative threading assembly refinement server (I-TASSER; https://zhanggroup.org/I-TASSER/) (accessed on 1 September 2025) [48]. The protein structure was prepared using Protein Repair and Analysis Server (PRAS; https://www.protein-science.com) (accessed on 1 September 2025) and AutoDock (version 1.5.7) tool protocol [49,50]. The protein and ligand pdbqt files for molecular docking were generated using the AutoDock tool. For the primary docking analysis, active-site docking was performed for the six targets with suitable co-crystallized ligand structures. Target-specific docking grids were centered on the corresponding co-crystallized ligands and configured to encompass the experimentally occupied binding pockets (Supplementary File S1 Table S4). Prior to BITC ligand docking, each protocol was evaluated by native-ligand redocking in three independent runs using fixed random seeds. Agreement with the crystallographic poses was assessed using symmetry-corrected heavy-atom RMSD calculated with sPyRMSD (version 0.9.0) [51,52]; RMSD ≤ 2.0 Å was considered near-native pose recovery. BITC was subsequently docked using the same receptor preparation, active-site grid, and Vina parameters (Supplementary File S1 Tables S4 and S5). The top-1 scores from three independent BITC docking runs were summarized as the mean ± standard deviation (SD). The VINA parameters for each receptor are outlined in Supplementary File S1 Table S4. The blind-docking analysis was performed only as an exploratory comparison and is provided in Supplementary File S1, Table S7, and was not used for the primary interpretation. All docking calculations were performed using AutoDock Vina (Windows version 1.2.7) [53,54]. The best docking scores were selected, and the generated output complex pose was computed through PyMol software (Windows version 3.0.3). The protein–ligand interaction analysis and generation of the three-dimensional conformation were performed using Discovery Studio Visualizer software (Dassault Systèmes BIOVIA, Discovery Studio 2024 Client Windows version 24.1.0; San Diego, CA, USA) or ChimeraX software (version 1.7.1).

### 2.5. In Silico Drug-likeness and ADMET Assessment

BITC was evaluated using SwissADME [55] for physicochemical properties, medicinal-chemistry alerts, and compliance with the Lipinski, Ghose, Veber, Egan, and Muegge filters. ADMETlab 3.0 [56] was used to predict absorption, distribution, metabolism, excretion, and toxicity endpoints. Binary probabilities ≥ 0.50 were interpreted as positive model predictions. All results were considered preliminary computational estimates rather than experimental evidence of safety or clinical suitability.

## 3. Results

### 3.1. Identification of Candidate BITC-Associated COVID-19 Targets

**Figure 2:**
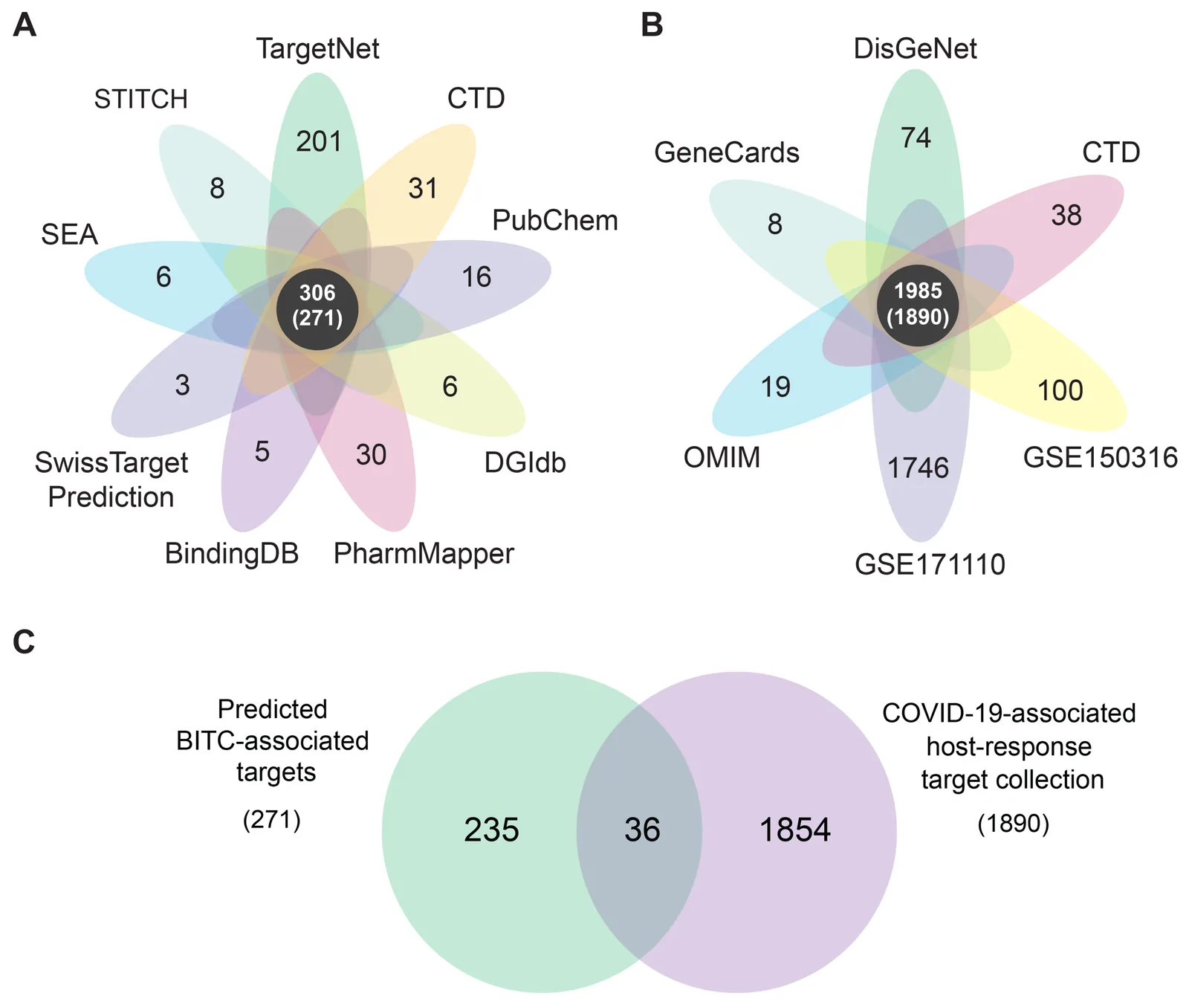
Identification of candidate overlapping targets between BITC-associated targets and COVID-19-associated targets. (**A**) Distribution of BITC-associated target entries retrieved from compound-target databases. A total of 306 pooled entries were obtained, yielding 271 unique targets after duplicate removal. (**B**) Distribution of COVID-19-associated target entries obtained from disease-gene databases and transcriptomic datasets. A total of 1985 pooled entries were collected, yielding 1890 unique targets after duplicate removal. (**C**) Venn diagram showing the intersection between 271 BITC-associated targets and 1890 COVID-19-associated targets, resulting in 36 overlapping candidate targets selected for downstream analyses.

A total of 306 BITC-associated target entries were retrieved from the selected databases, including TargetNet (201), CTD (31), PharmMapper (30), PubChem (16), STITCH (8), SEA (6), BindingDB (5), SwissTargetPrediction (3), and DGIdb (6). After removing duplicates, 271 unique BITC-associated targets were retained (Figure 2A). Differential expression analysis identified 100 DEGs from GSE150316 and 1746 DEGs from GSE171110, as shown by the normalized expression distributions and volcano plots (Supplementary Figure S1). For COVID-19-associated targets (Figure 2B), the integrated target pool consisted of 1890 unique COVID-19-related targets from DisGeNET (74), CTD (38), OMIM (19), and GeneCards (8). Finally, 36 candidate targets were determined as shown in the Venn diagram (Figure 2C; Supplementary File Table S1).

### 3.2. Construction of PPI Network and Identification of Hub Genes

**Figure 3:**
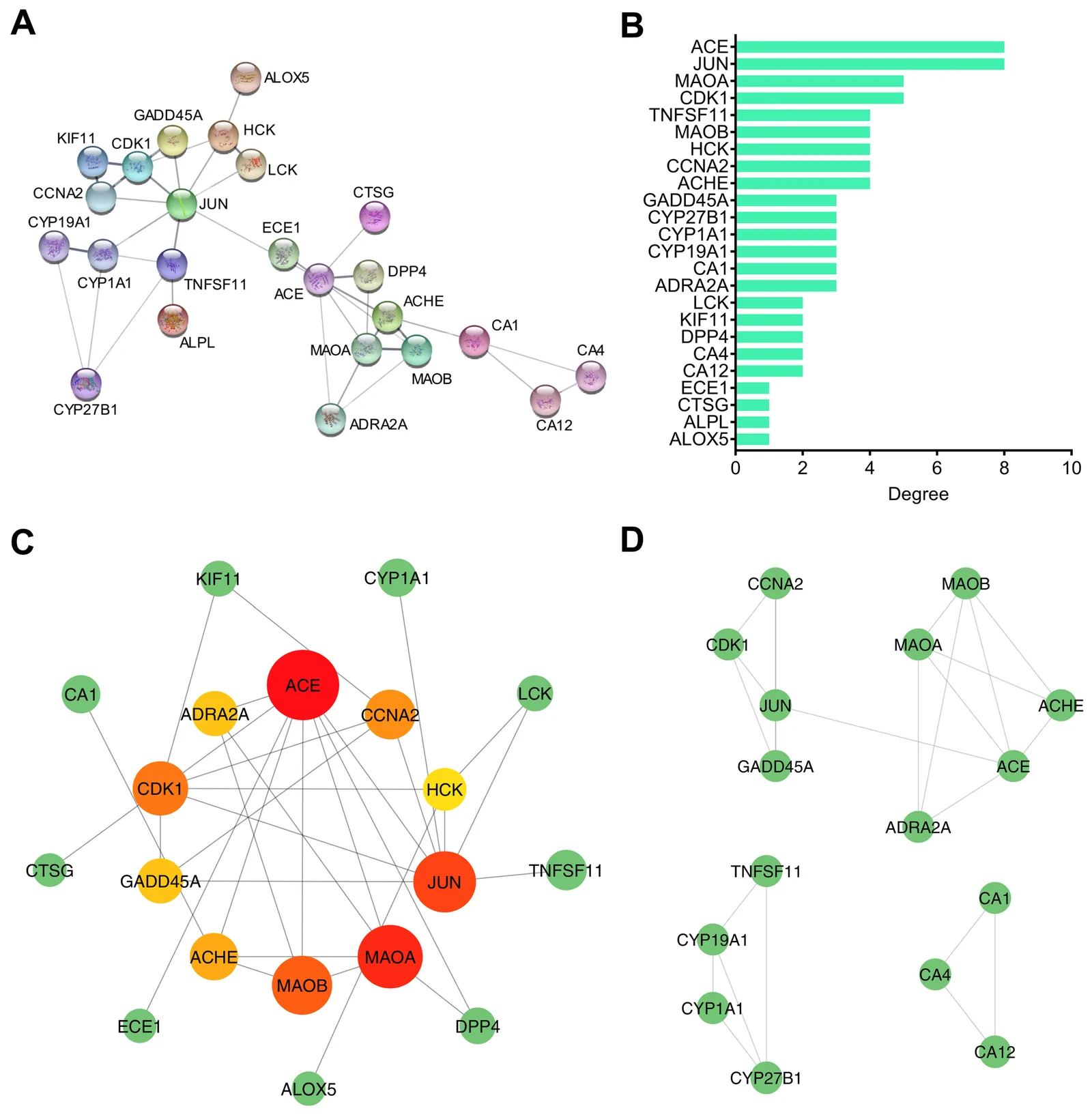
PPI network analysis and hub-gene candidates of BITC-associated COVID-19 targets. (**A**) PPI network of intersecting BITC and COVID-19 targets. (**B**) Network topology calculated the degree ranking; the y-axis represents the calculated network degree. (**C**) Top 10 hub genes using cytoHubba MCC algorithm. Green circles represent the nodes, and the gradient color of orange with different node sizes represents MCC scoring. (**D**) MCODE clustering analysis showing densely connected modules within the PPI network.

The 36 candidate BITC-associated COVID-19 targets were used to construct a PPI network (Figure 3A; Supplementary File S1 Figure S2), which contained 24 connected nodes and 39 interaction edges after removal of disconnected nodes (Figure 3A). Degree-based topology analysis showed that ACE and JUN had the highest degree values, followed by MAOA, CDK1, TNFSF11, MAOB, HCK, CCNA2, ACHE, and GADD45A (Figure 3B). Using MCC-based cytoHubba ranking, the top 10 hub genes were ACE, JUN, MAOA, CDK1, MAOB, HCK, CCNA2, ACHE, GADD45A, and ADRA2A (Figure 3C and Table 1). MCODE analysis identified densely connected modules within the PPI network, with the main cluster comprising nine nodes and 16 interaction edges (Figure 3D).

### 3.3. GO and KEGG Enrichment Analysis

**Figure 4:**
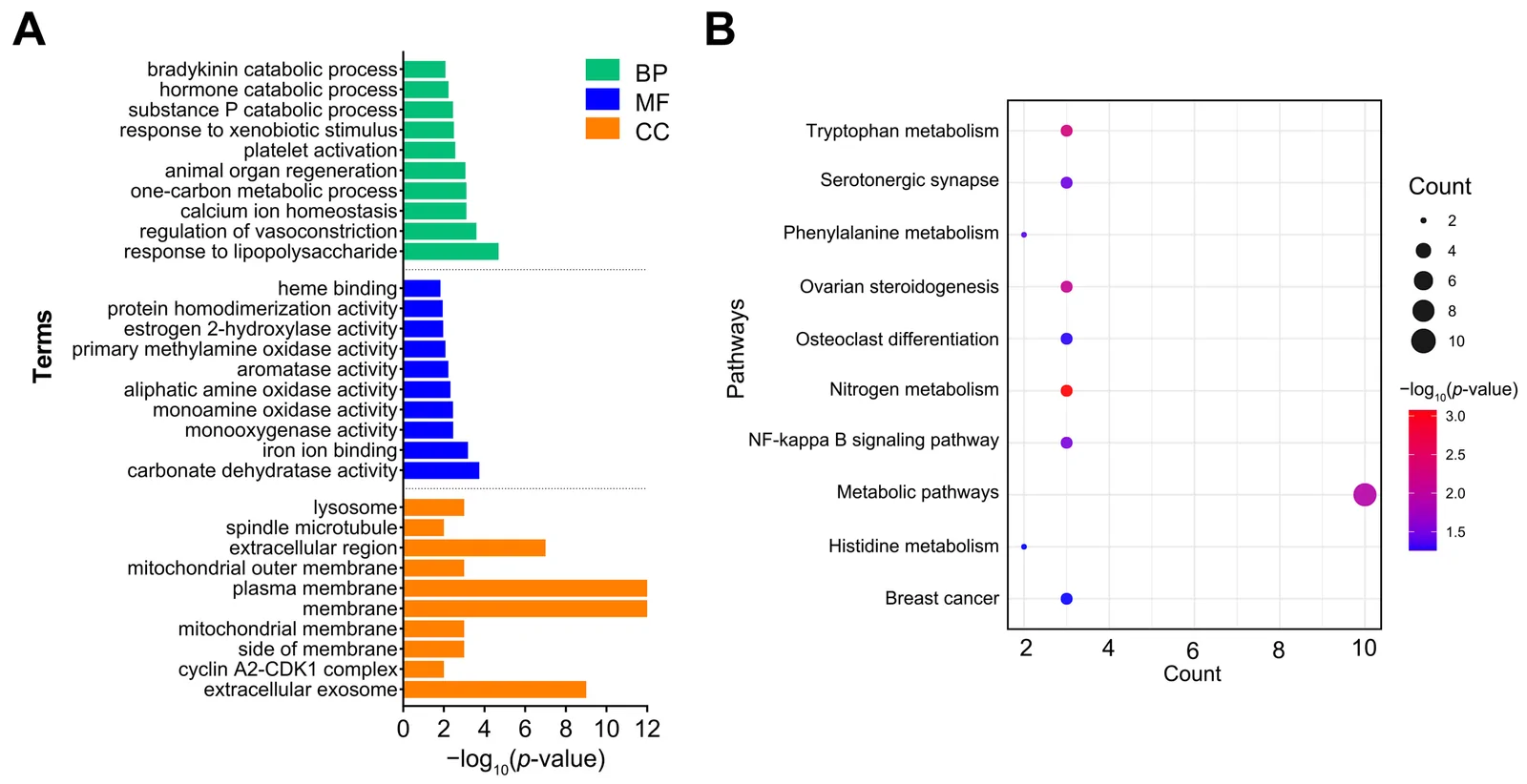
GO and KEGG enrichment analysis of candidate BITC-associated COVID-19 hub genes. (**A**) Gene Ontology enrichment analysis showing the top biological process (BP), molecular function (MF), and cellular component (CC) terms ranked by −log_10_ (raw *p*-value). (**B**) KEGG pathway enrichment analysis showing pathways ranked by nominal enrichment. Bubble size represents the number of associated genes, and color represents −log_10_ (raw *p*-value). Statistical significance after multiple-testing correction was defined as FDR < 0.05.

GO enrichment analysis (Figure 4A; Supplementary File S1 Table S2) of PPI hub genes showed significant enrichment in biological process (BP) related to the response to lipopolysaccharide (five genes; *p* = 2.50 × 10^−4^; FDR = 0.009344), regulation of vasoconstriction (three genes; *p* = 2.50 × 10^−4^; FDR = 0.057412), and calcium ion homeostasis (three genes; *p* = 7.75 × 10^−4^; FDR = 0.079521). Molecular function (MF) terms were mainly associated with carbonate dehydratase activity (three genes; *p* = 1.84 × 10^−4^; FDR = 0.028832), iron ion binding (four genes; *p* = 6.63 × 10^−4^; FDR = 0.052083), monooxygenase activity (three genes; *p* = 0.003499412; FDR = 0.140506), and monoamine oxidase activity (two genes; *p* = 0.003579763; FDR = 0.140506). Cellular component (CC) enrichment was predominantly linked to the plasma membrane (12 genes; *p* = 0.025584075; FDR = 0.394636), membrane (12 genes; *p* = 0.020088346; FDR = 0.394636), extracellular exosome (nine genes; *p* = 0.001993393; FDR = 0.118283), and extracellular region (seven genes; *p* = 0.035946029; FDR = 0.443018). KEGG pathway enrichment analysis showed that the hub genes were mainly associated with metabolic pathways (10 genes; *p* = 0.011715; FDR = 0.286044), NF-κB signaling pathway (three genes; *p* = 0.029513; FDR = 0.527247), nitrogen metabolism (12 genes; *p* = 8.43 × 10^−4^; FDR = 0.107884), osteoclast differentiation (three genes; *p* = 0.051758; FDR = 0.527247), serotonergic synapse (three genes; *p* = 0.034889; FDR = 0.527247), and tryptophan metabolism (three genes; *p* = 0.005132; FDR = 0.249007) (Figure 4B; Supplementary File S1 Table S3). Among these, the NF-κB signaling pathway was identified as a key immune–inflammatory pathway represented within the enriched KEGG terms.

### 3.4. Molecular Docking Analysis of BITC with Candidate Hub Targets

**Figure 5:**
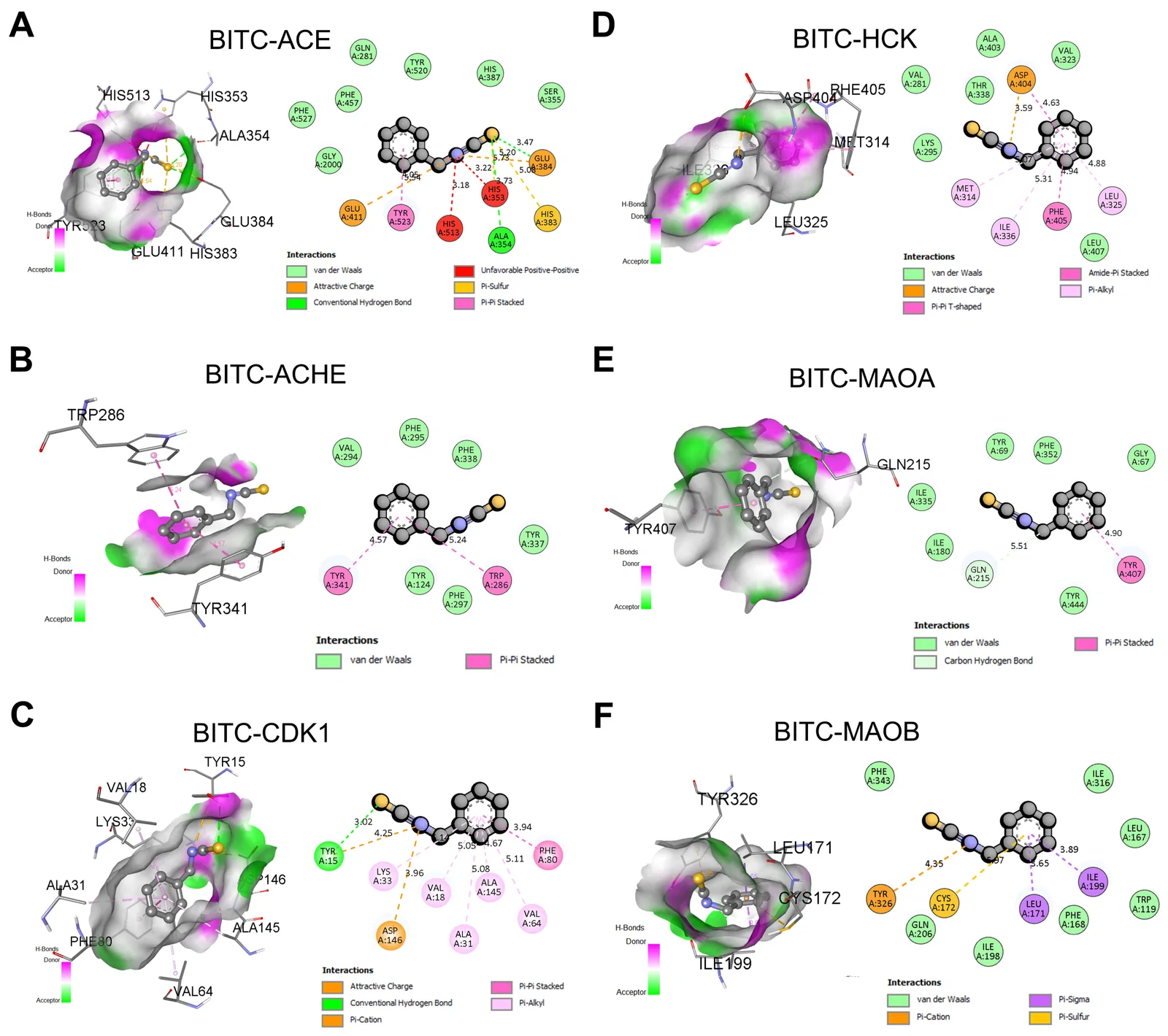
Predicted active-site binding poses and residue interactions of BITC with (**A**) ACE, (**B**) ACHE, (**C**) CDK1, (**D**) HCK, (**E**) MAOA, and (**F**) MAOB. The left panel represents the top-ranked BITC pose with three-dimensional hydrogen-bond donor/acceptor surface, and the right panel illustrates the two-dimensional interaction diagram and predicted contact distances.

Active-site-directed molecular docking was performed for the six MCC-prioritized hub targets with available co-crystallized ligands. The predicted BITC poses are presented in Figure 5, the Vina scores are summarized in Table 2, and the detailed residue interactions are provided in Supplementary File S1 Table S6. The mean docking scores ranged from −5.380 to −6.437 kcal/mol. CDK1 produced the most negative score (−6.437 kcal/mol), followed by MAOA (−5.973 kcal/mol), MAOB (−5.783 kcal/mol), ACHE (−5.603 kcal/mol), HCK (−5.543 kcal/mol), and ACE (−5.380 kcal/mol). The predicted BITC-target complexes showed variable interaction profiles (Figure 5), including conventional and carbon–hydrogen contacts, π-π, π–alkyl, π–sulfur, π–cation, π–sigma, charge-associated interactions, and van der Waals interactions. The BITC-ACE complex formed conventional hydrogen bonds with Glu384 and Ala354, whereas BITC-CDK1 formed a conventional hydrogen bond with Tyr15. MAOA showed a carbon–hydrogen contact with Gln215 and a π-π contact with Tyr407, whereas MAOB exhibited π–cation, π–sulfur, and π–sigma contacts involving Tyr326, Cys172, Leu171, and Ile199. Van der Waals contacts were observed across most complexes. Exploratory blind-docking results are provided in Supplementary File S1 (Figure S3 and Table S8).

### 3.5. Predicted Drug-likeness and ADMET Profile of BITC

BITC showed no violations of the Lipinski, Veber, or Egan filters, whereas two Ghose and one Muegge violations were recorded. SwissADME predicted high gastrointestinal absorption and non-substrate behavior toward P-glycoprotein. ADMETlab 3.0 predicted 93.69% plasma protein binding, a 6.96% unbound fraction, plasma clearance of 7.71 mL/min/kg, and an elimination half-life of 1.53 h. Positive model classifications were obtained for P-glycoprotein inhibition, five cytochrome P450 inhibition endpoints, drug-induced liver injury, Ames mutagenicity, skin sensitization, and respiratory toxicity. Platform-dependent absorption, distribution, cardiac-channel, and negative toxicity classifications are reported in Table 3 and Supplementary File S1 Table S9.

## 4. Discussion

This study provides a hypothesis-generating in silico assessment of benzyl isothiocyanate from *S. persica* in relation to COVID-19-associated host-response targets. By integrating predicted compound targets with COVID-19-associated transcriptomic and disease-related data, followed by PPI network analysis, functional enrichment, and molecular docking, the present study identified candidate molecular associations that may help guide further investigation of BITC within the context of COVID-19 host responses. Similar systems-level approaches combining transcriptomic, network-based, and structural analyses have been used to prioritize candidate targets and pathways associated with natural bioactive compounds in COVID-19-related studies [57,58,59].

The identification of 36 overlapping targets indicated that a defined subset of predicted BITC-associated targets intersects with COVID-19-associated host-response genes. These overlapping targets formed the basis for subsequent network and functional analyses. Comparable natural product-based network pharmacology studies have used compound-related targets, disease-associated genes, PPI topology, GO/KEGG enrichment, and molecular docking to refine broad target sets into more interpretable candidate molecular networks [59,60,61,62]. In the present study, this approach prioritized a group of targets spanning vascular/RAS-related biology, inflammatory regulation, monoamine metabolism, and cell-cycle-associated processes, suggesting that the predicted BITC–COVID-19 association is not restricted to a single molecular pathway.

The PPI identified ACE, JUN, MAOA, CDK1, MAOB, HCK, CCNA2, ACHE, GADD45A, and ADRA2A as the principal hub targets within the predicted network. The prioritization of ACE is consistent with evidence implicating ACE/RAS-related pathways in COVID-19 pathophysiology [63]. ACE provides a link to renin–angiotensin system biology, which has been implicated in the vascular, inflammatory, and cardiovascular manifestations of COVID-19. JUN is relevant to the network context because it has been identified as a hub target in COVID-19-related herbal network pharmacology analyses involving cytokine- and immune-associated pathways [64]. As a component of the AP-1 transcriptional complex, JUN also provides a connection to inflammatory and stress-responsive signaling. The prioritization of MAOA added a monoamine-related component, supported by computational evidence linking monoamine oxidase enzymes with SARS-CoV-2-associated neurological contexts and by broader evidence linking MAO activity with inflammatory regulation [65,66]. The prioritization of MAOA and MAOB therefore introduces a less commonly emphasized monoamine-related component to the predicted network. Experimental evidence is stronger for MAOB, as the SARS-CoV-2 spike protein has been reported to interact with MAOB and increase its activity, with associated mitochondrial dysfunction and oxidative stress [67]. In contrast, MAOA should be interpreted more cautiously as a network-prioritized candidate rather than an established COVID-19 regulator. Additional experimental studies have linked SARS-CoV-2 infection with CDK1-associated cell-cycle dysregulation and have implicated cyclin A2 (CCNA2) in viral replication [68,69]. ACHE may similarly represent a cholinergic-associated host-response component, as altered ACHE expression has been reported in critically ill COVID-19 patients [70]. Together, these findings suggest that the predicted BITC-associated network may involve interconnected vascular, inflammatory, and neuroimmune-related host processes rather than a single dominant target.

The enrichment analysis results further supported this multidimensional interpretation and identified biological themes related to innate inflammatory response, vascular regulation, calcium homeostasis, monoamine oxidase activity, and metabolic pathway organization. The enrichment of responses to lipopolysaccharide and NF-κB signaling is most consistent with an innate immune–inflammatory interpretation, as NF-κB has been described as a central pathway in SARS-CoV-2-associated inflammatory and cytokine-related host responses [71]. This interpretation is also biologically relevant to BITC, as experimental studies have reported effects of BITC on inflammatory signaling pathways, including MAPK/NF-κB and inflammasome-related mechanisms [71,72]. These observations do not establish that BITC modulates the same pathways in COVID-19 but provide biological context for the predicted associations identified in the present analysis.

Enriched terms related to vasoconstriction and calcium ion homeostasis are consistent with reported endothelial/RAS-related dysfunction and mineral homeostasis abnormalities in the context of COVID-19 [73,74]. The enrichment of monoamine oxidase activity, serotonergic synapses, and tryptophan metabolism further suggests a metabolic and neuroimmune-associated host-response component, consistent with reports of kynurenine and tryptophan–kynurenine pathway alterations in acute, long-, and post-COVID-19 settings [75,76]. Although these enriched terms should not be interpreted as direct evidence of BITC activity in these processes, their convergence with the prioritized network targets supports the hypothesis that the predicted BITC-associated target profile spans several interconnected features of the COVID-19 host response.

Molecular docking provided a supplementary structural layer for prioritizing selected candidate targets. Following native-ligand validation, active-site-directed docking was restricted to ACE, ACHE, CDK1, HCK, MAOA, and MAOB. The resulting docking scores and predicted interaction profiles were considered comparative indicators of possible ligand accommodation within experimentally defined binding sites rather than evidence of stable binding, inhibition, or target modulation. Although CDK1 showed the most negative mean docking score among the evaluated targets, differences in docking scores were not used to establish biological target priority. The biological relevance of the identified targets was interpreted primarily from network, enrichment, and available disease-related evidence.

The predicted docking scores were considered comparative structural indicators rather than definitive measures of binding affinity because docking outcomes are inherently influenced by scoring function performance, receptor preparation, binding-site definition, and protocol-specific parameters [77]. Accordingly, the predicted poses and scores primarily support target prioritization within the present computational framework rather than confirmation of direct molecular binding.

This study applied a complementary in silico workflow that integrated target prediction, COVID-19-associated data mining, PPI network analysis, GO/KEGG enrichment, and molecular docking to evaluate the predicted BITC-associated target–pathway relationships. The strength of this approach is that it allowed predicted BITC-associated relationships to be examined at the levels of target overlap, network topology, functional enrichment, and structural interaction. However, these findings remain dependent on database coverage, prediction algorithms, dataset selection, and docking assumptions. This study did not include molecular dynamics simulations to assess the temporal stability and conformational persistence of the predicted BITC-protein complexes, MM/PBSA or MM/GBSA binding free-energy calculations, or experimental assays to assess the stability, binding characteristics, or biological consequences of the predicted interactions. Therefore, the predicted targets, hub genes, enriched pathways, and docking poses should be considered hypothesis-generating outputs that require experimental validation and further structural assessment, including molecular dynamics and binding free-energy analyses.

In addition, BITC showed drug-likeness compatibility, although high plasma protein binding, short predicted half-life, and cytochrome P450 classifications indicate potential exposure and metabolic-interaction liabilities. Reported variability in isothiocyanate bioavailability and metabolism, together with the differing platform outputs, limits direct extrapolation to biological exposure [78]. The predicted liver injury, mutagenicity, sensitization, and respiratory-toxicity alerts require experimental assessment and should inform compound prioritization rather than biological or clinical interpretation [79].

Overall, the predicted BITC-associated targets intersect with molecular processes related to immune–inflammatory signaling, vascular/RAS-associated biology, monoamine-related activity, and metabolic regulation. Rather than identifying a single dominant mechanism, the integrated analysis suggests a potential multi-target host-response profile that warrants further investigation. These computational findings provide a theoretical framework for prioritizing specific BITC-target relationships for future mechanistic and experimental studies.

## 5. Conclusions

In conclusion, this in silico study identified potential BITC-associated targets overlapping with COVID-19-related host-response pathways, particularly those involved in immune–inflammatory, vascular/RAS-related, monoamine-associated, and metabolic processes. These findings provide a theoretical basis for further investigation of benzyl isothiocyanate from *Salvadora persica* as a predicted multi-target candidate associated with COVID-19-associated host-response pathways. Experimental validation is required to confirm the biological relevance of these computational findings.

## Figures and Tables

**Table 1:** MCC top 10 BITC and COVID-19 PPI-related targets.

UniProt ID	Gene Name	Description
P12821	ACE	Angiotensin-converting enzyme
P05412	JUN	Transcription factor Jun
P21397	MAOA	Amine oxidase flavin-containing A
P06493	CDK1	Cyclin-dependent kinase 1
P08913	ADRA2A	Alpha-2A adrenergic receptor
P27338	MAOB	Amine oxidase flavin-containing B
P08631	HCK	Tyrosine-protein kinase HCK
P20248	CCNA2	Cyclin-A2
P22303	ACHE	Acetylcholinesterase
P24522	GADD45A	Growth arrest and DNA damage-inducible protein GADD45 alpha

**Table 2:** Co-crystallized ligand redocking validation and active-site-directed docking scores of BITC with MCC-prioritized hub targets.

Receptor	Mean Co-Crystallized Ligand RMSD ± SD (Å)	BITC Score (kcal/mol)
ACE	1.039 ± 0.128	−5.380
ACHE	1.514 ± 0.101	−5.603
CDK1	0.648 ± 0.021	−6.437
HCK	0.314 ± 0.003	−5.543
MAOA	1.044 ± 0.015	−5.973
MAOB	0.634 ± 0.021	−5.783

**Table 3:** Predicted physicochemical, drug-likeness, and ADMET profile of benzyl isothiocyanate.

Category	Parameter	Predicted Value or Classification
Physicochemical	Molecular weight	149.21 g/mol
	Consensus LogP	3.09
	Topological polar surface area	44.45 Å^2^
	Predicted aqueous solubility	−3.07 log mol/L; soluble
	Hydrogen-bond acceptors/donors	1/0
	Rotatable bonds	2
Drug-likeness	Lipinski filter	Pass; 0 violations
	Veber filter	Pass; 0 violations
	Egan filter	Pass; 0 violations
	Ghose filter	Fail; 2 violations
	Muegge filter	Fail; 1 violation
	Bioavailability score	0.55
	PAINS/Brenk alerts	0/2
Absorption	Gastrointestinal absorption	High
	Caco-2 permeability	−4.51 log cm/s
	P-glycoprotein substrate	No; ADMETlab score = 0.0002
	P-glycoprotein inhibitor	Positive model classification; score = 0.967
Distribution	Plasma protein binding	93.69%
	Fraction unbound	6.96%
	BBB permeation	Yes; negative ADMETlab classification, score = 0.054
Metabolism	CYP1A2 inhibitor	Positive model classification; score = 0.930
	CYP2C19 inhibitor	Positive model classification; score = 0.953
	CYP2C9 inhibitor	Positive model classification; score = 0.576
	CYP2D6 inhibitor	Positive model classification; score = 0.697
	CYP3A4 inhibitor	Positive model classification; score = 0.567
Excretion	Plasma clearance	7.71 mL/min/kg
	Half-life	1.53 h
Toxicity	hERG inhibition	Endpoint-dependent: general score = 0.080, negative; 10 µM score = 0.942, positive
	Drug-induced liver injury	Positive model classification; score = 0.999
	Ames mutagenicity	Positive model classification; score = 0.864
	Skin sensitization	Positive model classification; score > 0.999
	Respiratory toxicity	Positive model classification; score > 0.999
	Carcinogenicity	Negative model classification; score = 0.024
	Human hepatotoxicity	Negative model classification; score = 0.005

## Data Availability

The original contributions presented in this study are included in the article. Further inquiries can be directed to the corresponding author.

## Supplementary Materials

The following supporting information can be downloaded at https://www.mdpi.com/article/10.3390/cimb48080849/s1.
