# Supplementary material for: In Silico and Molecular Docking Analysis of Benzyl Isothiocyanate from Salvadora persica as a Predicted Multi-Target Candidate for COVID-19 Host Responses
Source: Curr Issues Mol Biol. 2026 Aug 21;48(8):849. doi: 10.3390/cimb48080849 (PMC13511586; doi:10.3390/cimb48080849)
Supplement: Supplementary file 1 [file cimb-48-00849-s001.zip › cimb-4469130-supplementary.pdf]

Supplementary File S1

Figure S1. Differential expression analysis of COVID-19-related GEO datasets

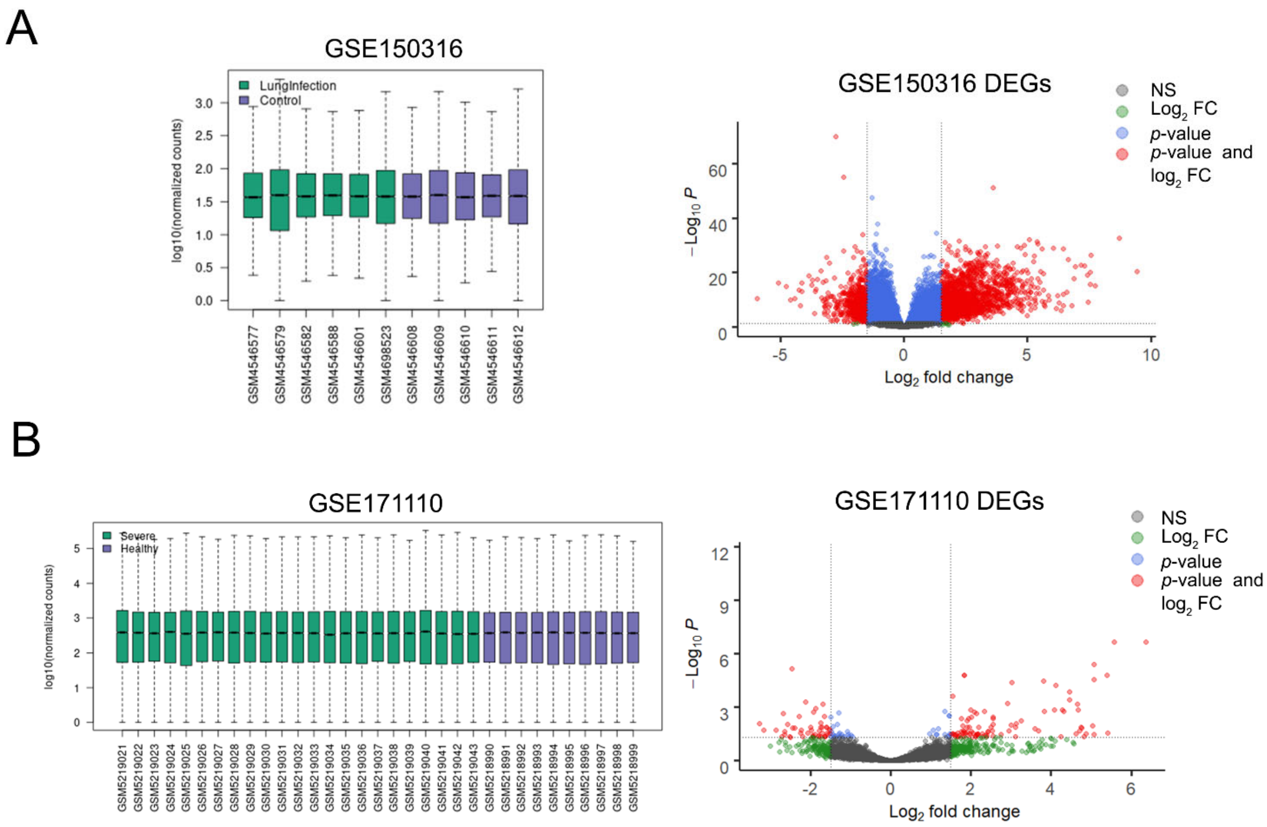

Differential expression analysis of GSE150316 and GSE171110. Boxplots show (left) the normalized expression distributions across samples in each dataset, and volcano plots (right) display the differentially expressed genes identified between COVID-19-related and control groups. DEGs from both datasets were pooled and used for downstream COVID19-related targets construction.

Figure S2. Sensitivity analysis of the STRING PPI network at higher confidence thresholds

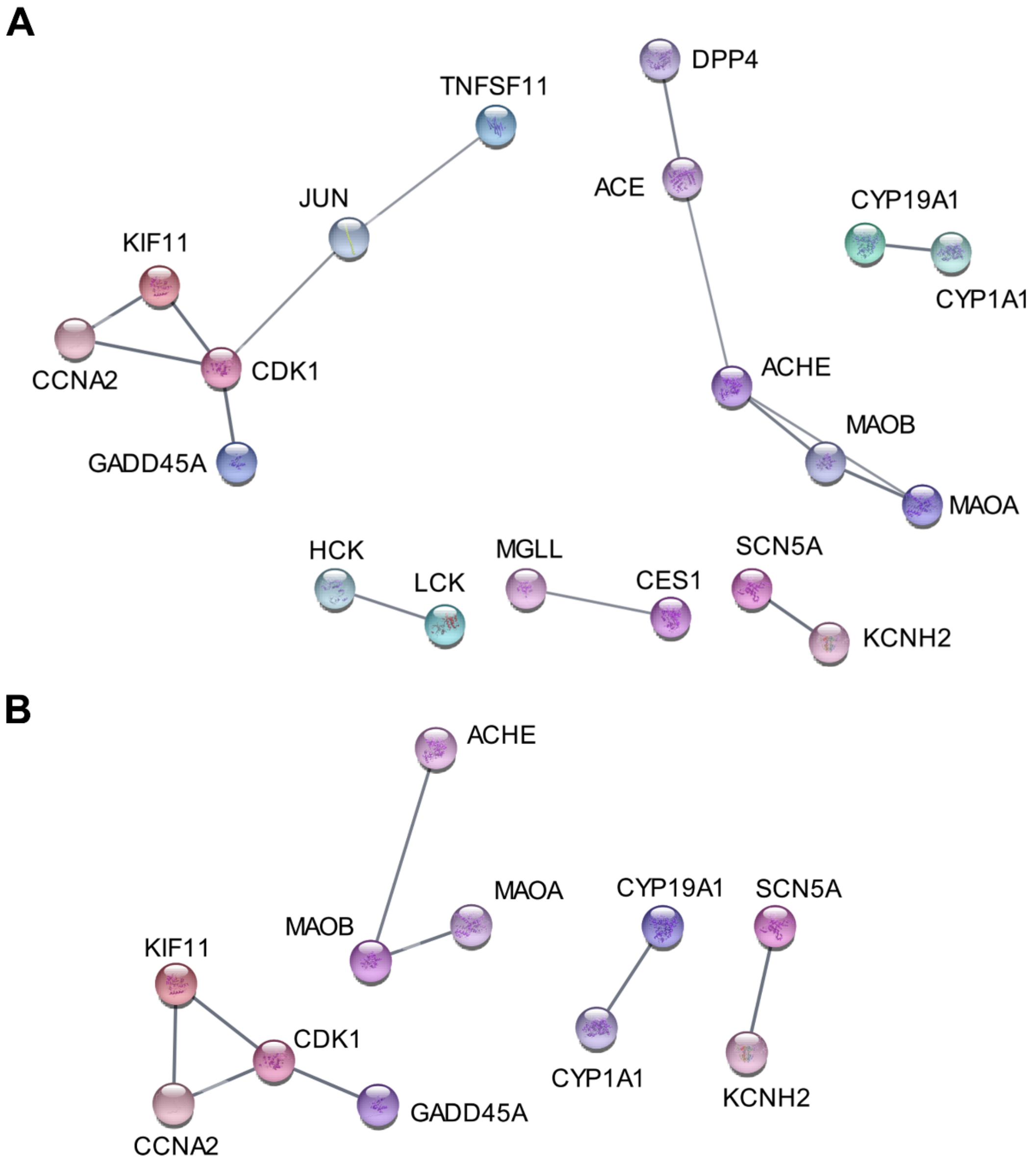

STRING network sensitivity analysis at higher interaction-confidence thresholds. Protein-protein interaction networks were reconstructed using STRING confidence scores of (A) 0.7 (high confidence) and (B) 0.9 (highest confidence). Increasing the confidence threshold reduced network connectivity and excluded weaker associations. Only genes retaining at least one interaction at each threshold are displayed; disconnected genes were omitted from the visualization. Nodes represent target proteins, and edges represent STRING-supported functional associations; edge colors indicate the underlying evidence sources.

Figure S3. Exploratory blind-docking poses of BITC with the MCC-prioritized targets.

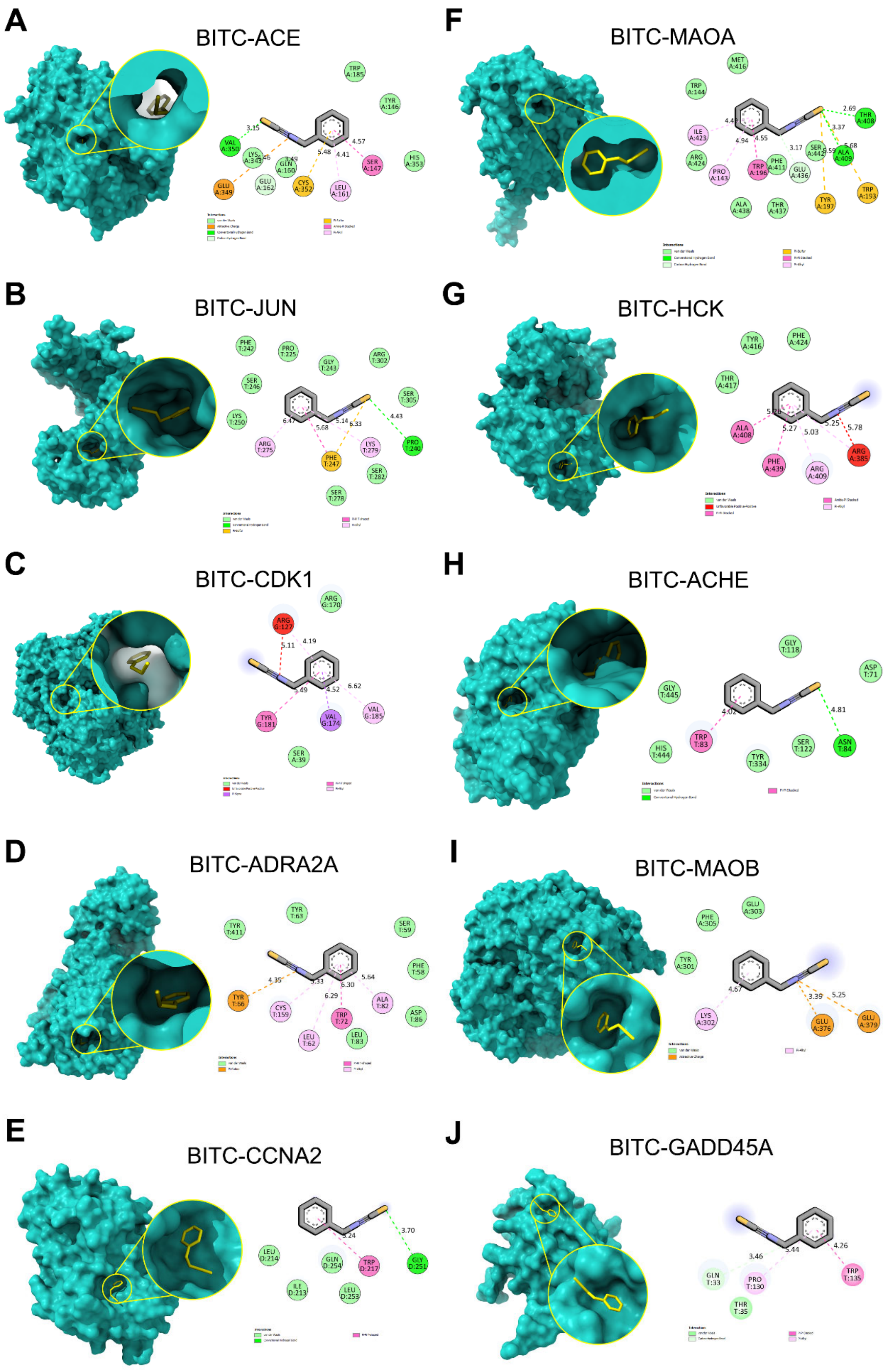

Exploratory blind-docking poses of BITC with the MCC-prioritized targets. Predicted docking poses of BITC with (A) ACE, (B) JUN, (C) CDK1, (D) ADRA2A, (E) CCNA2, (F) MAOA, (G) HCK, (H) ACHE, (I) MAOB, and (J)

GADD45A complex analysis. The left panel represents the three-dimensional receptor and binding pose of the ligand, and the right panel illustrates the receptor-ligand complex interaction. The detailed VINA parameters and docking results are shown in Table S7 and Table S8.

Table S1. Identified common related targets of BITC and COVID-19

ACE, ACHE, ADRA2A, ALPL, ALOX5, CA1, CA12, CA4, CCNA2, CDK1, CES1, CTSG, CYP19A1, CYP1A1, DPEP1, DPP4, ECE1, GADD45A, GSTT1, HCK, HRH2, JUN, KCNH2, KIF11, LCK, MAOA, MAOB, MGLL, OPRL1, ORM2, PLIN5, RORA, SCN5A, TGM3, TNFSF11, VDR

Table S2. List of top 10 enriched GO terms.

**Category**	**GO**	**Term**	**Count**	***p*-value**	**FDR**
BP	GO:0032496	response to lipopolysaccharide	5	2.04×10^{-05}	0.009344
	GO:0019229	regulation of vasoconstriction	3	2.50×10^{-04}	0.057412
	GO:0055074	calcium ion homeostasis	3	7.75×10^{-04}	0.079521
	GO:0006730	one-carbon metabolic process	3	7.75×10^{-04}	0.079521
	GO:0031100	animal organ regeneration	3	8.66×10^{-04}	0.079521
	GO:0030168	platelet activation	3	0.00273248	0.203019
	GO:0009410	response to xenobiotic stimulus	4	0.003235515	0.203019
	GO:0010814	substance P catabolic process	2	0.003538458	0.203019
	GO:0042447	hormone catabolic process	2	0.005890774	0.300429
	GO:0010815	bradykinin catabolic process	2	0.008237777	0.378114
MF	GO:0004089	carbonate dehydratase activity	3	1.84×10^{-04}	0.028832
	GO:0005506	iron ion binding	4	6.63×10^{-04}	0.052083
	GO:0004497	monooxygenase activity	3	0.003499412	0.140506
	GO:0097621	monoamine oxidase activity	2	0.003579763	0.140506
	GO:0052595	aliphatic amine oxidase activity	2	0.004770291	0.149787
	GO:0070330	aromatase activity	2	0.005959459	0.155939
	GO:0008131	primary methylamine oxidase activity	2	0.008333717	0.186913
	GO:0101021	estrogen 2-hydroxylase activity	2	0.010702551	0.198142
	GO:0042803	protein homodimerization activity	5	0.011358433	0.198142
	GO:0020037	heme binding	3	0.014762001	0.231763
CC	GO:0070062	extracellular exosome	9	0.001993393	0.118283
	GO:0097122	cyclin A2-CDK1 complex	2	0.0022109	0.118283
	GO:0098552	side of membrane	3	0.008094667	0.28871
	GO:0031966	mitochondrial membrane	3	0.019823537	0.394636
	GO:0016020	membrane	12	0.020088346	0.394636
	GO:0005886	plasma membrane	12	0.025584075	0.394636
	GO:0005741	mitochondrial outer membrane	3	0.025817302	0.394636
	GO:0005576	extracellular region	7	0.035946029	0.443018
	GO:0005876	spindle microtubule	2	0.042278972	0.443018
	GO:0005764	lysosome	3	0.04775441	0.443018

Table S3. List of top 10 enriched KEGG pathways.

Term	Count	*p*-value	FDR
Nitrogen metabolism	3	8.43×10^{-04}	0.107884
Tryptophan metabolism	3	0.005132	0.249007
Ovarian steroidogenesis	3	0.007781	0.249007
Metabolic pathways	10	0.011715	0.286044
NF-kappa B signaling pathway	3	0.029513	0.527247
Serotonergic synapse	3	0.034889	0.527247
Phenylalanine metabolism	2	0.040494	0.527247
Osteoclast differentiation	3	0.051758	0.527247
Breast cancer	3	0.055029	0.527247
Histidine metabolism	2	0.055271	0.527247

Table S4. Redocking active-site Vina parameters

Protein	Protein PDB ID	Co-crystallized ligand PDB code	Box XYZ coordinates	Set box size XYZ (Å)	Exhaustiveness	Modes	Energy range (kcal/mol)
ACE	1O86	LPR	x: 41.014<br>y: 34.325<br>z: 46.441	17× 17 ×17	64	20	5
ACHE	8DT7	3VI	x: 90.498<br>y: 53.575<br>z: 4.190	10×10 × 10	64	20	5
CDK1	4Y72	LZ9	x: 29.215<br>y: -72.059<br>z: 184.798	21× 21 × 21	64	20	5
HCK	1QCF	PP1	x: 24.940<br>y: 30.222<br>z: 40.249	17× 17 × 17	64	20	5
MAOA	2Z5X	HRM	x: 40.582<br>y: 26.931<br>z: -14.540	12× 12 × 12	64	20	5
MAOB	1OJ9	1PB	x: 51.098<br>y: 157.555<br>z: 28.896	12× 12 × 12	64	20	5

Table S5. Co-crystallized ligands redocking results

Protein	Random Seed	Native-ligand RMSD (Å)	Pose rank	Docking score (kcal/mol)
ACE	20261	1.1250	1	-8.6779
	20262	0.8917	1	-8.8213
	20263	1.1017	1	-8.7107
ACHE	20261	1.6300	11	-5.7926
	20262	1.4474	10	-5.6762
	20263	1.4644	13	-5.6666

CDK1	20261	0.6701	1	-10.5991
	20262	0.6280	1	-10.5413
	20263	0.6465	1	-10.5467
HCK	20261	0.3164	2	-9.2985
	20262	0.3112	1	-9.2950
	20263	0.3151	2	-9.2653
MAOA	20261	1.0385	4	-8.0165
	20262	1.0335	3	-8.0090
	20263	1.0608	4	-8.0308
MAOB	20261	0.6294	8	-8.5240
	20262	0.6157	11	-8.6589
	20263	0.6576	10	-8.5330

Table S6. Predicted interactions of BITC within the validated receptor active sites

Affinity (kcal/mol)	Affinity (kcal/mol)	Total H-bonds	Residue - Length (Å) - Type
BITC-ACE	-5.380 ± 0.000	2	GLU384 - 3.47 (Conventional hydrogen bond)<br>ALA354 - 3.73 (Conventional hydrogen bond)<br>GLU411 - 5.54 (Attractive charge)<br>GLU384 - 5.08 (Attractive charge)<br>HIS383 - 5.73 (Pi-sulfur)<br>TYR523 - 4.05 (Pi-Pi stacked)<br>HIS353 - 3.22 (Unfavorable positive-positive)<br>HIS513 - 3.18 (Unfavorable positive-positive)<br><br>GLN281, SER355, HIS387, PHE457, TYR520, PHE527, and GLY2000 (van der Waals)
BITC-ACHE	-5.603 ± 0.025	0	TYR341 - 4.57 (Pi-Pi stacked)<br>TRP286 - 5.24 (Pi-Pi stacked)<br><br>TYR124, VAL294, PHE295, PHE297, PHE338, and TYR337 (van der Waals)
BITC-CDK1	-6.437 ± 0.012	1	TYR15 - 3.02 (Conventional hydrogen bond)<br>TYR15 - 4.25 (Pi-cation)<br>ASP146 - 3.96 (Attractive charge)<br>LYS33 - 4.14 (Pi-alkyl)<br>VAL18 - 5.05 (Pi-alkyl)<br>ALA31 - 5.08 (Pi-alkyl)<br>VAL64 - 5.11 (Pi-alkyl)<br>ALA145 - 4.67 (Pi-alkyl)<br>PHE80 - 3.94 (Pi-Pi stacked)
BITC-HCK	-5.543 ± 0.006	0	ASP404 - 3.59 (Attractive charge)<br>ASP404 - 4.63 (Pi-Pi T-shaped)<br>MET314 - 5.07 (Pi-alkyl)<br>ILE336 - 5.31 (Pi-alkyl)<br>PHE405 - 4.94 (Amide-Pi stacked)<br>LEU325 - 4.88 (Pi-alkyl)

			VAL281, LYS295, VAL323, THR338, ALA403, and LEU407 (van der Waals)
BITC-MAOA	-5.973 ± 0.006	0	GLN215 - 5.51 (Carbon-hydrogen contact)<br>TYR407 - 4.90 (Pi-Pi stacked)<br><br>GLY67, TYR69, ILE180, ILE335, PHE352, and TYR444 (van der Waals)
BITC-MAOB	-5.783 ± 0.006	0	TYR326 - 4.35 (Pi-cation)<br>CYS172 - 5.97 (Pi-sulfur)<br>LEU171 - 3.65 (Pi-sigma)<br>ILE199 - 3.89 (Pi-sigma)<br><br>TRP119, LEU167, PHE168, ILE198, GLN206, ILE316, and PHE343 (van der Waals)

Table S7. Whole protein blind-docking VINA parameters.

Ligand & receptor	Box XYZ coordinates	Set box size XYZ (Å)	Exhaust-iveness	Modes	Energy range (kcal/mol)	Grid space
BITC-ACE	x: 40.649<br>y: 37.362<br>z: 43.459	126 × 126 ×126	128	40	15	0.500
BITC-JUN	x: 0.21<br>y: -0.21<br>z: -0.633					0.500
BITC-CDK1	x: -14.322<br>y: 12.344<br>z: -22.279					0.700
BITC-ADRA2A	x: -1.534<br>y: -3.182<br>z: 3.88					0.600
BITC-CCNA2	x: -27.194<br>y: 45.445<br>z: 25.669					0.400
BITC-MAOA	x: 34.696<br>y: 28.13<br>z: -20.095					0.700
BITC-HCK	x: 12.709<br>y: 29.767<br>z: 32.383					0.600
BITC-ACHE	x: 0.91<br>y: -0.713<br>z: -1.356					0.500

BITC-MAOB	x: 37.656<br>y: 138.851<br>z: 20.388					0.600
BITC-GADD45A	x: -0.372<br>y: 0.098<br>z: -0.166					0.500

Table S8. Whole protein blind-docking scores.

BITC-receptor complex	Affinity (kcal/mol)	Total H-Bonds	Residue - Length (Å) - Type
BITC-ACE	-5.0	1	VAL350 - 3.15 (Hydrogen bond)<br>GLN160 - 3.49 (Carbon H-bonds)<br>GLU349 - 4.46 (Attractive Charge)<br>CYS352 - 5.48 (Pi-Sulfur)<br>LEU161 - 4.41 (Pi-Alkyl)<br>SER147 - 4.57 (Amide-Pi-Stacked)<br>TRP185 - (vdW)<br>HIS353 - (vdW)<br>TYR146 - (vdW)
BITC-JUN	-5.4	1	PRO240 - 4.43 (Hydrogen bond)<br>LYS279 - 5.14 (Pi-Alkyl)<br>PHE247 - 6.33 (Pi-Sulfur)<br>PHE247 - 5.68 (Pi-Pi T-shaped)<br>ARG275 - 6.47 (Pi- Alkyl)<br>LYS250 - (vdW)<br>SER246 - (vdW)<br>PHE242 - (vdW)<br>PRO225 - (vdW)<br>GLY243 - (vdW)<br>ARG302 - (vdW)<br>SER35 - (vdW)<br>SER282 - (vdW)<br>SER278 - (vdW)
BITC-CDK1	-5.1	0	VAL185 - 6.62 (Pi-Alkyl)<br>ARG127 - 4.19 (Pi-Alkyl)<br>ARG127 - 5.11 (Unfavorable Positive-Positive)<br>VAL174 - 6.62 (Pi-Sigma)<br>TYR181 - 5.49 (Pi-Pi T-shaped)<br>SER39 - (vdW)<br>ARG170 - (vdW)
BITC-CCNA2	-5.5	1	GLY251 - 3.70 (Hydrogen bond)<br>TRP217 - 5.24 (Pi-Pi T-shaped)<br>LEU - (vdW)<br>ILE213 - (vdW)<br>GLN254 - (vdW)<br>LEU253 - (vdW)
BITC-MAOA	-6.0	2	THR408 - 2.69 (Hydrogen bond)<br>ALA409 - 3.37 (Hydrogen bond)

			GLU436 - 3.17 (Carbon Hydrogen bond)<br>TRP196 - 4.55 (Pi-Pi Stacked)<br>PRO143 - 4.94 (Pi-Alkyl)<br>ILE423 - 4.94 (Pi-Alkyl)<br>TRP193 - 5.68 (Pi-Sulfur)<br>TYR197 - 5.59 (Pi-Sulfur)<br>MET416 - (vdW)<br>TRP144 - (vdW)<br>ARG424 - (vdW)<br>ALA438 - (vdW)<br>THR437 - (vdW)<br>PHE411 - (vdW)<br>SER422 - (vdW)
BITC-HCK	-5.3	0	ALA408 - 5.26 (Pi-Pi Stacked)<br>PHE439 - 5.27 (Pi-Pi Stacked)<br>ARG409 - 5.03 (Pi-Alkyl)<br>ARG385 - 5.25 (Pi-Alkyl)<br>ARG385 - 5.78 (Unfavorable Positive-Positive)<br>THR417 - (vdW)<br>TYR416 - (vdW)<br>PHE424 - (vdW)
BITC-ADRA2A	-5.4	0	TYR66 - 4.53 (Pi-Cation)<br>CYS159 - 5.33 (Pi-Alkyl)<br>LEU62 - 6.29 (Pi-Alkyl)<br>ALA82 - 5.64 (Pi-Alkyl)<br>TRP72 - 6.30 (Pi-Pi T-shaped)<br>TYR411 - (vdW)<br>TYR63 - (vdW)<br>SER59 - (vdW)<br>PHE58 - (vdW)<br>ASP86 - (vdW)<br>LEU83 - (vdW)
BITC-ACHE	-5.7	1	ASN84 - 4.81 (Hydrogen bond)<br>TRP83 - 4.02 (Pi-Pi Stacked)<br>GLY118 - (vdW)<br>ASP71 - (vdW)<br>SER122 - (vdW)<br>TYR334 - (vdW)<br>HIS444 - (vdW)<br>GLY445 - (vdW)
BITC- MAOB	-4.5	0	LYS302 - 4.67 (Pi-Alkyl)<br>GLU376 - 3.39 (Attractive Charge)<br>GLU379 - 5.25 (Attractive Charge)<br>GLU303 - (vdW)<br>PHE305 - (vdW)<br>TYR301 - (vdW)
BITC- GADD45A	-4.2	0	GLN33 - 3.46 (Carbon Hydrogen Bond)<br>PRO130 - 5.44 (Pi-Alkyl)<br>TRP135 - 4.26 (Pi-Pi Stacked)

Table S9. Cross-platform physicochemical, drug-likeness, and ADMET predictions for benzyl isothiocyanate

Category	Endpoint	SwissADME	ADMETlab 3.0	Cross-platform assessment
Physicochemical properties	Molecular weight	149.21 g/mol	149.03 g/mol	Consistent across platforms
	Octanol-water partition coefficient	3.09	2.97	Consistent across platforms
	Octanol-water distribution coefficient at pH 7.4	Not reported	3.03	Platform-specific result
	Predicted aqueous solubility	-3.07 log mol/L	-3.04 log mol/L	Generally consistent
	Topological polar surface area	44.45 Å²	12.36 Å²	Descriptor discrepancy requiring verification
	Hydrogen-bond acceptors/donors	1/0	1/0	Consistent across platforms
	Rotatable bonds	2	2	Consistent across platforms
Drug-likeness	Lipinski filter	Pass, 0 violations	Pass, 0 violations	Consistent across platforms
	Veber filter	Pass, 0 violations	Not reported	SwissADME only
	Egan filter	Pass, 0 violations	Not reported	SwissADME only
	Ghose filter	Fail, 2 violations	Not reported	SwissADME only
	Muegge filter	Fail, 1 violation	Not reported	SwissADME only
	Bioavailability score	0.55	Not reported	SwissADME only
	Pan-assay interference alerts	0	0	Consistent across platforms
	Brenk structural alerts	2	Not reported	SwissADME only
Absorption	Gastrointestinal absorption	High	Model output: 0.001	Inconsistent across platforms
	Caco-2 cell permeability	Not reported	-4.511 log cm/s	Continuous estimate
	P-glycoprotein substrate	No	0.0002, negative	Concordant
	P-glycoprotein inhibitor	Not reported	0.967, positive	Platform-specific result
Distribution	Blood-brain barrier permeation	Yes	0.054, negative	Inconsistent across platforms
	Plasma protein binding	Not reported	93.69%	Platform-specific result
	Fraction unbound in plasma	Not reported	6.96%	Platform-specific result
	Steady-state volume of distribution	Not reported	0.049 log L/kg	Platform-specific result
Metabolism	Cytochrome P450 1A2 inhibition	Unavailable	0.930, positive	Platform-specific result
	Cytochrome P450 2C19 inhibition	Unavailable	0.953, positive	Platform-specific result
	Cytochrome P450 2C9 inhibition	Unavailable	0.576, positive	Platform-specific result
	Cytochrome P450 2D6 inhibition	Unavailable	0.697, positive	Platform-specific result

	Cytochrome P450 3A4 inhibition	Unavailable	0.567, positive	Platform-specific result
Excretion	Plasma clearance	Not reported	7.71 mL/min/kg	Platform-specific result
	Elimination half-life	Not reported	1.53 h	Platform-specific result
Toxicity	Human ether-à-go-go-related gene channel inhibition	Not reported	0.080, negative	Platform-specific result
	Human ether-à-go-go-related gene channel inhibition at 10 µM	Not reported	0.942, positive	Endpoint-dependent result
	Drug-induced liver injury	Not reported	0.999, positive	Model alert
	Ames mutagenicity	Not reported	0.864, positive	Model alert
	Skin sensitization	Not reported	>0.999, positive	Model alert
	Respiratory toxicity	Not reported	>0.999, positive	Model alert
	Carcinogenicity	Not reported	0.024, negative	Model classification
	Human hepatotoxicity	Not reported	0.005, negative	Model classification
	Drug-induced neurotoxicity	Not reported	0.503, borderline positive	Near classification threshold

Note: Values were generated using the same standardized neutral structure of benzyl isothiocyanate. For binary ADMETlab 3.0 endpoints, model outputs of 0.50 or higher were classified as positive and values below 0.50 as negative. These classifications represent computational model outputs rather than experimentally confirmed effects or absence of risk. Numerical outputs were compared across platforms only when the endpoint definitions were sufficiently similar. Inconsistent and platform-specific results were retained without preferential selection. “Not reported” indicates that the platform did not provide the endpoint, whereas “unavailable” indicates that the prediction could not be generated at the time of analysis.
